# Supplementary material for: Integrative genomics of the mammalian alveolar macrophage response to intracellular mycobacteria
Source: BMC Genomics. 2021 May 12;22:343. doi: 10.1186/s12864-021-07643-w (PMC8117616; doi:10.1186/s12864-021-07643-w)
Supplement: Supplementary file 8 — Additional file 8: S1 Fig. Principal component analysis (PCA) plots for individual animal bAM gene expression data at a 2 hpi, b 6 hpi, c 24 hpi, and d 48 hpi. S2 Fig. Gene ontology (GO) enrichment for functional modules identified from the differential co-expression correlation network generated from M. bovis-infected bAM gene expression at 24 hpi. S3 Fig. Gene ontology (GO) enrichment for functional modules identified from the differential co-expression correlation network generated from M. bovis-infected bAM gene expression at 48 hpi. S4 Fig. Venn diagram illustrating the overlaps for the three 24 hpi input gene sets used for integration with cattle GWAS data sets. S5 Fig. Venn diagram illustrating the overlaps for the three 48 hpi input gene sets used for integration with cattle GWAS data sets. RNA-seq statistics and results for this study are available at the Dryad Digital Repository: https://doi.org/10.5061/dryad.83bk3j9q6. [file 12864_2021_7643_MOESM8_ESM.docx]

**Supplementary Figures**

**
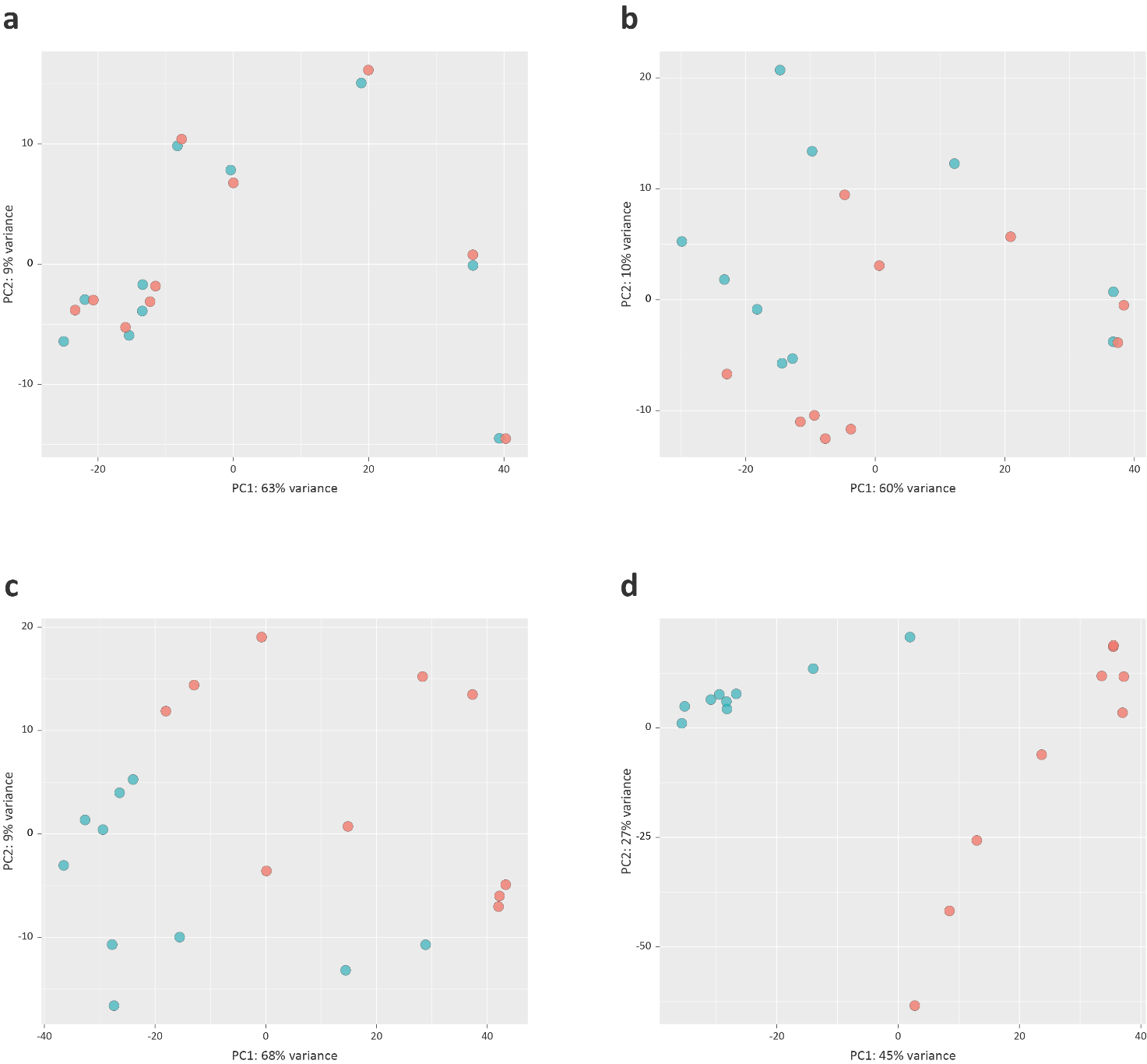
**

**S1 Fig.** Principal component analysis (PCA) plots for individual animal bAM gene expression data at **a** 2 hpi, **b** 6 hpi, **c** 24 hpi, and **d** 48 hpi. Red indicates *M. bovis*-infected animals and blue indicates non-infected control animals.

**
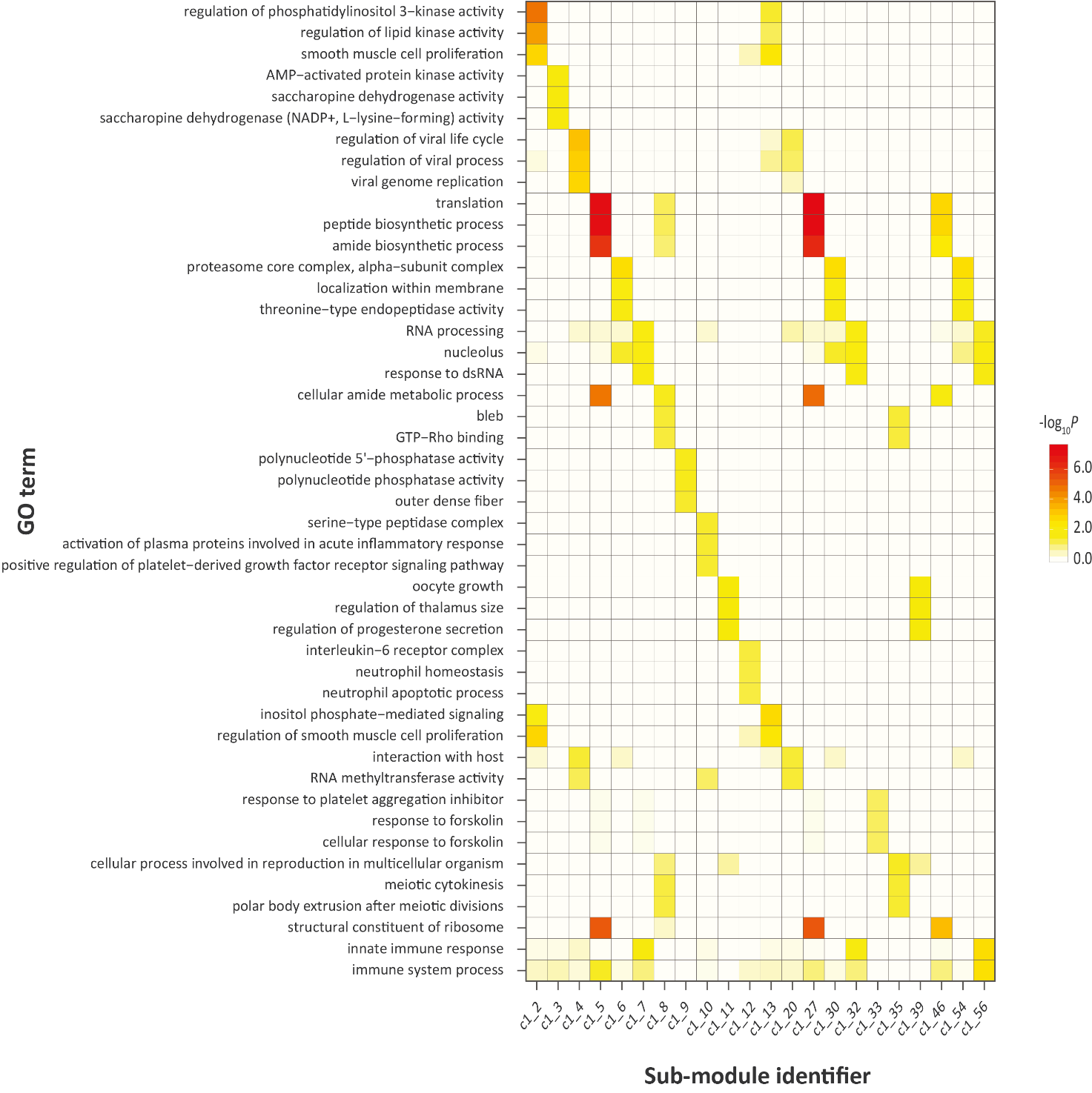
**

**S2 Fig.** Gene ontology (GO) enrichment for functional modules identified from the differential co-expression correlation network generated from *M. bovis*-infected bAM gene expression at 24 hpi.

**
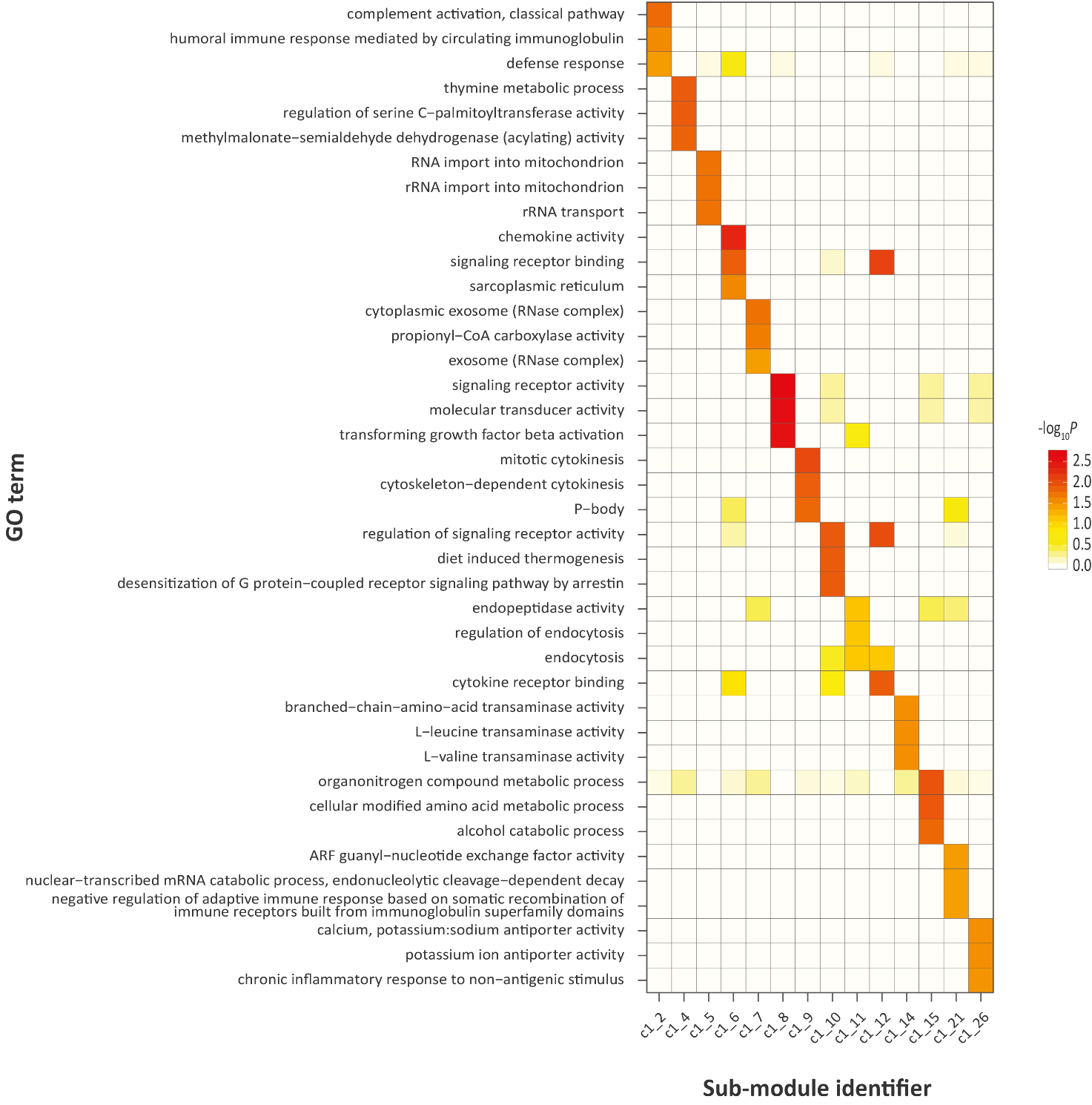
**

**S3 Fig.** Gene ontology (GO) enrichment for functional modules identified from the differential co-expression correlation network generated from *M. bovis*-infected bAM gene expression at 48 hpi.

**
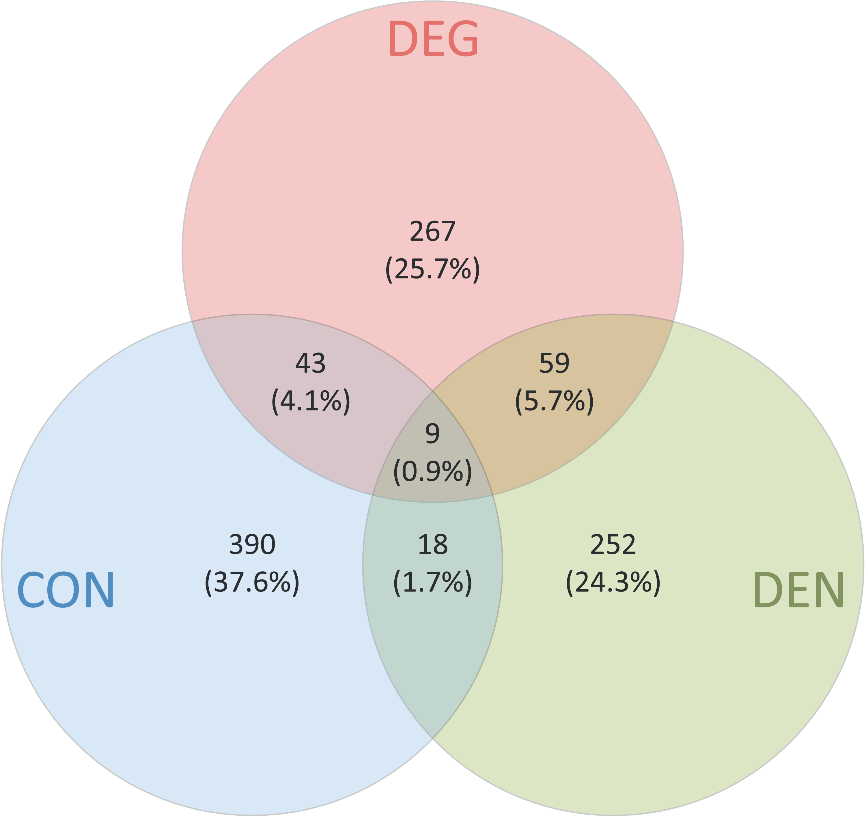
**

**S4 Fig.** Venn diagram illustrating the overlaps for the three 24 hpi input gene sets used for integration with cattle GWAS data sets.


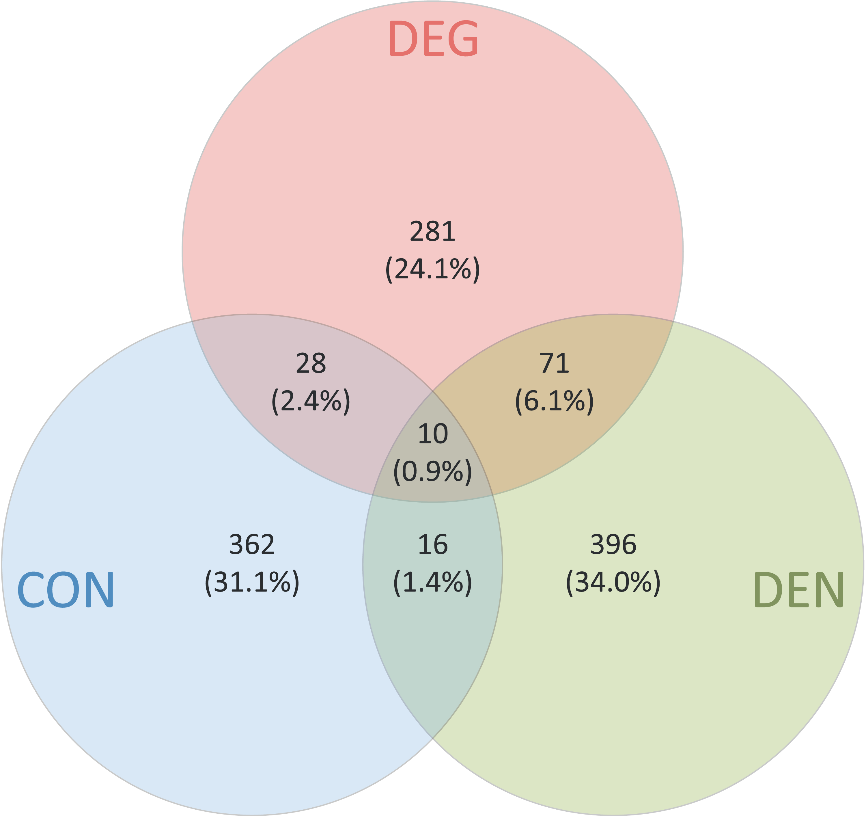


**S5 Fig.** Venn diagram illustrating the overlaps for the three 48 hpi input gene sets used for integration with cattle GWAS data sets.
